# Prognostic ability of mid-term worsening renal function after percutaneous coronary intervention: findings from the SHINANO registry

**DOI:** 10.1007/s00380-021-01837-8

**Published:** 2021-04-07

**Authors:** Yoshiteru Okina, Takashi Miura, Keisuke Senda, Minami Taki, Masanori Kobayashi, Masafumi Kanai, Yukari Okuma, Takashi Yanagisawa, Naoto Hashizume, Kyuhachi Otagiri, Kyoko Shoin, Noboru Watanabe, Soichiro Ebisawa, Kenichi Karube, Hiroyuki Nakajima, Tatsuya Saigusa, Yusuke Miyashita, Daisuke Kashiwagi, Keisuke Machida, Naoyuki Abe, Takahiro Tachibana, Yusuke Kanzaki, Takuya Maruyama, Hidetomo Nomi, Takahiro Sakai, Hisanori Yui, Tomoaki Mochidome, Takahiro Kobayashi, Toshio Kasai, Uichi Ikeda, Koichiro Kuwahara

**Affiliations:** 1 Department of Cardiology, Nagano Municipal Hospital, 1333-1 Tomitake, Nagano, 381-0006 Japan; 2 Department of Cardiovascular Medicine, Shinshu University School of Medicine, Nagano, Japan; 3 Department of Cardiology, Shinshu Ueda Medical Center, Ueda, Japan; 4 Department of Cardiology, Matsumoto Kyoritsu Hospital, Matsumoto, Japan; 5 Department of Cardiology, Nagano Red Cross Hospital, Nagano, Japan; 6 Epartment of Cardiology, Suwa Red Cross Hospital, Suwa, Japan; 7 Department of Cardiology, Saku Central Hospital, Saku, Japan; 8 Department of Cardiology, Shinonoi General Hospital, Nagano, Japan; 9 Department of Cardiology, Ina Central Hospital, Ina, Japan; 10 Department of Cardiology, Aizawa Hospital, Matsumoto, Japan; 11 Department of Cardiology, Okaya Municipal Hospital, Okaya, Japan; 12 Department of Cardiology, Hokushin General Hospital, Nagano, Japan; 13 Department of Cardiology, Matsushiro General Hospital, Nagano, Japan

**Keywords:** Prognostic factor, Major adverse cardiovascular event (MACE), Renal insufficiency, Coronary vascular disease

## Abstract

Chronic kidney disease is a prognostic factor for cardiovascular disease. Worsening renal function (WRF), specifically, is an important predictor of mortality in patients with acute myocardial infarction undergoing primary percutaneous coronary intervention (PCI). We evaluate the prognostic impact of mid-term WRF after PCI on future cardiovascular events. We examined the renal function data of 1086 patients in the first year after PCI using the SHINANO 5-year registry. Patients were divided into two groups, mid-term WRF and non-mid-term WRF, and primary outcomes were major adverse cardiovascular events (MACE) and death. Mid-term WRF was defined as an increase in creatinine (≥ 0.3 mg/dL) in the first year after PCI. Mid-term WRF was found in 101 patients (9.3%), and compared to non-mid-term WRF, it significantly increased the incidence of MACE (*p* < 0.001), and all-cause death (*p* < 0.001), myocardial infarction (*p* = 0.001). Furthermore, mid-term WRF patients had higher incidence of future heart failure (*p* < 0.001) and new-onset atrial fibrillation (*p* = 0.01). Patients with both mid-term WRF and chronic kidney disease had increased MACE compared to patients with either condition alone (*p* < 0.001). Similarly, patients with mid-term WRF and acute kidney injury had increased MACE compared to patients with either condition alone (*p* < 0.001). Multivariate Cox regression analysis revealed mid-term WRF as a strong predictor of MACE (hazard ratio: 2.50, 95% confidence interval 1.57–3.98, *p* < 0.001). Mid-term WRF after PCI negatively affects MACE, as well as future admission due to heart failure and new-onset atrial fibrillation, chronic kidney disease, and acute kidney injury.

## Introduction

Chronic kidney disease (CKD) is considered as an important prognostic factor in patients with coronary vascular disease, and the risk of cardiovascular events increased progressively with the estimated glomerular filtration rate (eGFR) below 60 mL/min/1.73 m^2^ of body-surface area: the adjusted hazard ratio for mortality was 1.4 times in an eGFR of 45–59 mL/min/1.73 m^2^ group, 2.0 in an eGFR of 30–44 mL/min/1.73 m^2^ group, 2.8 in an eGFR of 15–29 mL/min/1.73 m^2^ group, and 3.4 in an eGFR of less than 15 mL/min/1.73 m^2^ group [1]. Specifically, eGFR at baseline and in-hospital worsening renal function (WRF) are important predictors of mortality in patients with acute myocardial infarction (MI) undergoing primary percutaneous coronary intervention (PCI) [2]. Mortality in patients with an eGFR less than 45 mL/min/1.73 m^2^ has been shown to be 2.6 times higher compared to normal eGFR patients, and low eGFR and WRF were, respectively, independent predictor of all-cause mortality [2]. WRF is a powerful predictor for in-hospital mortality and coronary vascular complication in acute coronary syndrome patients [3]. In a previous study, in-hospital mortality for WRF patients was 18 times greater than non-WRF patients, and the risk for cardiovascular events was 4.5 times greater [3]. In another study, during 4-year follow-up, it is also said that WRF in the acute phase of patients with acute MI affects long-term prognosis, and WRF was independently associated with a higher risk of death [4]. The prognostic impact of mid-term WRF has not yet been fully characterized, despite this existing knowledge. To this end, we evaluated the relationship between long-term future cardiovascular events and mid-term WRF after PCI.

## Materials and methods

### Study design and participants

**Fig. 1:**
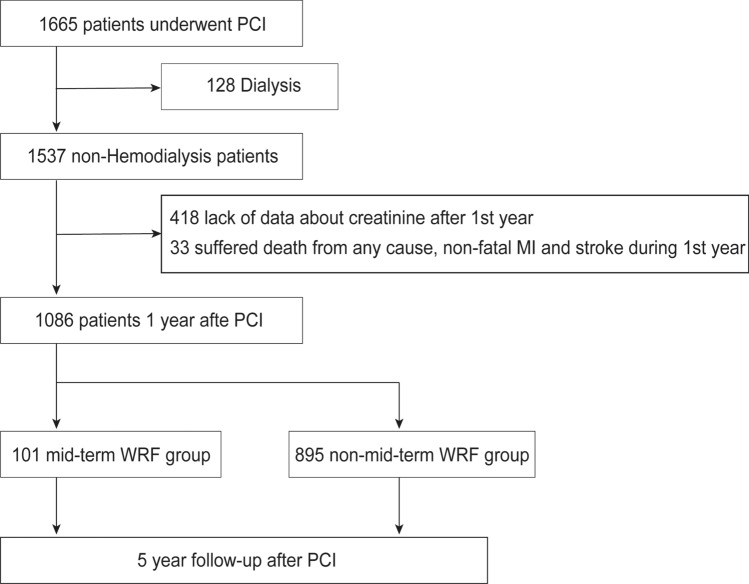
Patient selection flow chart. *PCI* percutaneous coronary intervention, *MI* myocardial infarction, *WRF* worsening renal function

This retrospective cohort study used data obtained from the Shinshu prospective multicenter analysis for elderly patients with coronary artery disease undergoing percutaneous coronary intervention (SHINANO) 5-year registry, a prospective, multicenter, observational registry designed to compare differences in baseline characteristics and short- and long-term outcomes after initial PCI between elderly and non-elderly patients. This was an all-comer registry with no exclusion criteria. Patients were prospectively followed for 5 years after enrollment. We examined a cohort of 1665 consecutive patients who underwent primary PCI for any coronary artery disease in the SHINANO 5-year registry between August 2012 and July 2013. We excluded 128 patients on dialysis, 418 patients with incomplete creatinine data 1 year after PCI, and 33 suffered death from any cause, non-fatal MI and stroke during 1st year. The remaining 1086 PCI patients with 1-year follow-up renal function data were divided into two groups: WRF (101 patients) and non-WRF (985 patients) (Fig. 1). The present study was approved by each of the hospitals’ ethics committees and was performed in accordance with the Declaration of Helsinki. The SHINANO 5-year registry is registered with the University Hospital Medical Information Network Clinical Trials Registry (UMIN-CTR), as accepted by the International Committee of Medical Journal Editors (No. UMIN 000010070).

### Outcome measure and definitions

The primary endpoint was defined as major adverse cardiovascular event (MACE), including all-cause death, non-fatal MI, and stroke after the first year post-PCI. The secondary endpoint was defined as future hospital admission due to heart failure, new induction of hemodialysis, and new-onset atrial fibrillation (AF). The survival analysis was started 1 year after PCI. These outcomes were ascertained through medical records and follow-up questionnaires sent to patients’ primary physician. Mid-term WRF was defined as an increase in creatinine ≥ 0.3 mg/dL at 6 months before and after 1 year after PCI, and CKD was defined as eGFR ≤ 60 mL/min/1.73 m^2^. Acute kidney injury (AKI) was defined worsened renal function during the in-hospital period, and each hospital made the determination to continue hospitalization or discharge the patient.

### Statistical analysis

Continuous variables are expressed as mean ± standard deviation, and categorical variables are expressed as numbers and percentages. Continuous variables were compared using the Student’s *t* test, and categorical variable were analyzed with the χ^2^ test. The cumulative incidence of MACE was estimated based on the Kaplan–Meier method, and differences were assessed using the log-rank test. Interaction analyses were performed using the Cox proportional hazards model. Multivariate logistic analysis was used to determine the significance of WRF as a predictor. In this study, any covariates that were significant in univariate analysis (*p* < 0.05) were assessed by multivariable analysis. A 2-tailed *p* value < 0.05 was considered significant.

## Results

Mean follow-up time was 1548 days. Mid-term WRF was found in 101 patients (9.3%), and this group was older, had lower baseline renal function, and were more likely to have a previous history of heart failure, cerebral infarction. However, contrast volume during PCI was similar between both the groups (Table 1).

**Table 1:** Clinical characteristics of patients at baseline

	All patients	WRF (+)	WRF (−)	*p*
Age	70.3 ± 10.3	73.2 ± 9.8	70.0 ± 10.3	0.002
Height (m)	1.61 ± 0.1	1.59 ± 0.1	1.61 ± 0.09	0.07
Body weight (kg)	62.3 ± 11.8	60.2 ± 13.1	62.5 ± 11.7	0.06
Body mass index (kg/m^2^)	23.8 ± 3.4	23.6 ± 3.4	23.9 ± 3.5	0.42
Number of males	851 (78.4)	73 (72.3)	778 (79.0)	0.22
Total contrast volume (mL)	169.0 ± 81.3	163.7 ± 81.0	169.5 ± 81.3	0.57
ST-elevation MI	275 (26.5)	37 (37.8)	238 (25.3)	0.009
Non-ST-elevation MI	44 (4.2)	3 (3.1)	41 (4.4)	0.39
Unstable angina pectoris	113 (10.9)	11 (11.2)	102 (10.9)	0.91
History of MI	293 (27.0)	28 (37.7)	265 (26.9)	0.86
History of cardiac surgery	59 (5.5)	6 (6.1)	53 (5.5)	0.78
History of CABG	86 (7.9)	13 (12.9)	73 (7.4)	0.05
History of heart failure	139 (12.8)	31 (30.7)	108 (11.0)	< 0.001
History of cerebral hemorrhage	10 (0.9)	2 (1.2)	8 (0.8)	0.24
History of cerebral infarction	89 (8.2)	14 (13.9)	75 (7.6)	0.03
Peripheral artery disease	104 (9.6)	11 (10.9)	93 (9.5)	0.64
AF	119 (11.0)	15 (14.9)	104 (10.6)	0.19
Hypertension	801 (73.8)	82 (81.2)	719 (73.0)	0.08
Dyslipidemia	670 (61.7)	66 (65.3)	604 (61.3)	0.43
Diabetes mellitus	378 (34.8)	43 (42.6)	335 (34.0)	0.09
At discharge				
Aspirin	1051 (97.8)	97 (97.0)	654 (97.8)	0.39
Thienopyridine	977 (90.9)	89 (89.0)	888 (91.1)	0.49
Cilostazol	30 (2.8)	3 (3.0)	27 (2.8)	0.54
Eicosapentaenoic acid	50 (4.7)	2 (1.3)	48 (4.9)	0.14
Warfarin	124 (11.6)	16 (16.0)	108 (11.1)	0.14
Dabigatran	21 (2.0)	2 (2.0)	19 (2.0)	0.59
Statin	828 (77.2)	78 (78.8)	750 (77.0)	0.69
Angiotensin-converting enzyme inhibiter	373 (34.9)	38 (38.4)	335 (34.5)	0.44
Angiotensin II receptor blockers	390 (36.5)	33 (33.3)	357 (36.8)	0.49
β-Blocker	458 (43.0)	58 (58.6)	400 (41.4)	0.001
Red blood cells (million/mm^3^)	484.5 ± 592.3	423.6 ± 75.0	490.7 ± 621.1	0.28
Hemoglobin (g/dL)	14.1 ± 6.1	14.0 ± 7.2	14.1 ± 6.0	0.92
Hematocrit (%)	41.1 ± 5.1	38.8 ± 6.7	41.3 ± 4.8	< 0.001
Blood urea nitrogen (mg/dL)	17.7 ± 6.2	19.5 ± 6.5	17.6 ± 6.1	0.003
Creatinine (mg/dL)	0.93 ± 0.4	1.1 ± 0.5	0.9 ± 0.4	0.003
eGFR (mL/min/1.73 m^2^)	56.0 ± 18.4	49.6 ± 19.1	56.5 ± 18.1	0.003
Total cholesterol (mg/dL)	178.8 ± 40.1	182.3 ± 45.1	178.5 ± 39.6	0.38
Triglycerides (mg/dL)	137.3 ± 96.9	142.2 ± 91.9	136.8 ± 97.4	0.59
High-density lipoprotein (mg/dL)	48.1 ± 13.5	41.2 ± 11.9	48.5 ± 13.5	0.003
Low-density lipoprotein (mg/dL)	106.0 ± 34.5	107.6 ± 40.5	105.9 ± 33.9	0.64
Glycated hemoglobin (%)	6.5 ± 6.1	6.6 ± 1.9	6.5 ± 6.3	0.87
Blood glucose (mg/dL)	140.8 ± 56.7	160.5 ± 76.8	138.9 ± 54.0	0.009
C-reactive protein (mg/dL)	0.7 ± 2.9	1.9 ± 7.6	0.5 ± 1.7	0.09

*MI* myocardial infarction, *CABG* coronary artery bypass graft, *AF* atrial fibrillation, *eGFR* estimated glomerular filtration rate

**Fig. 2:**
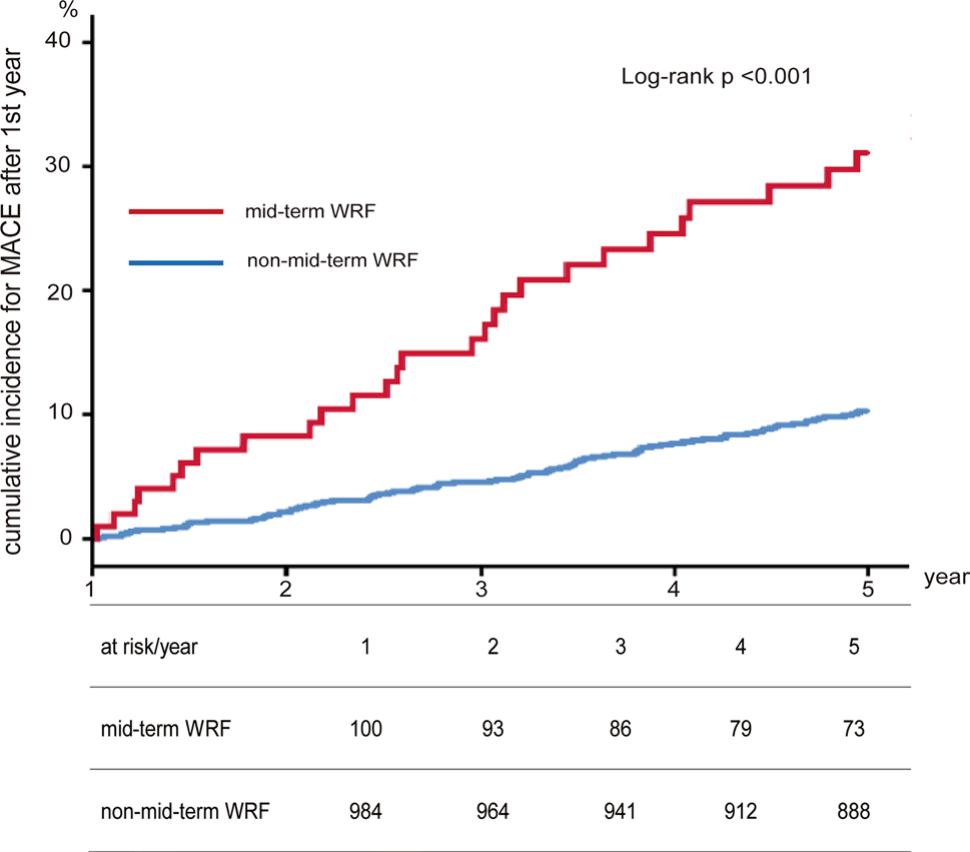
Cumulative incidence for MACE rate according to mid-term WRF. The incidences of MACE were significantly higher in mid-term WRF patients compared to non-mid-term WRF patients. MACE includes all-cause death, non-fatal myocardial infarction, and stroke. *MACE* major adverse cardiac event, *WRF* worsening renal function

**Fig. 3:**
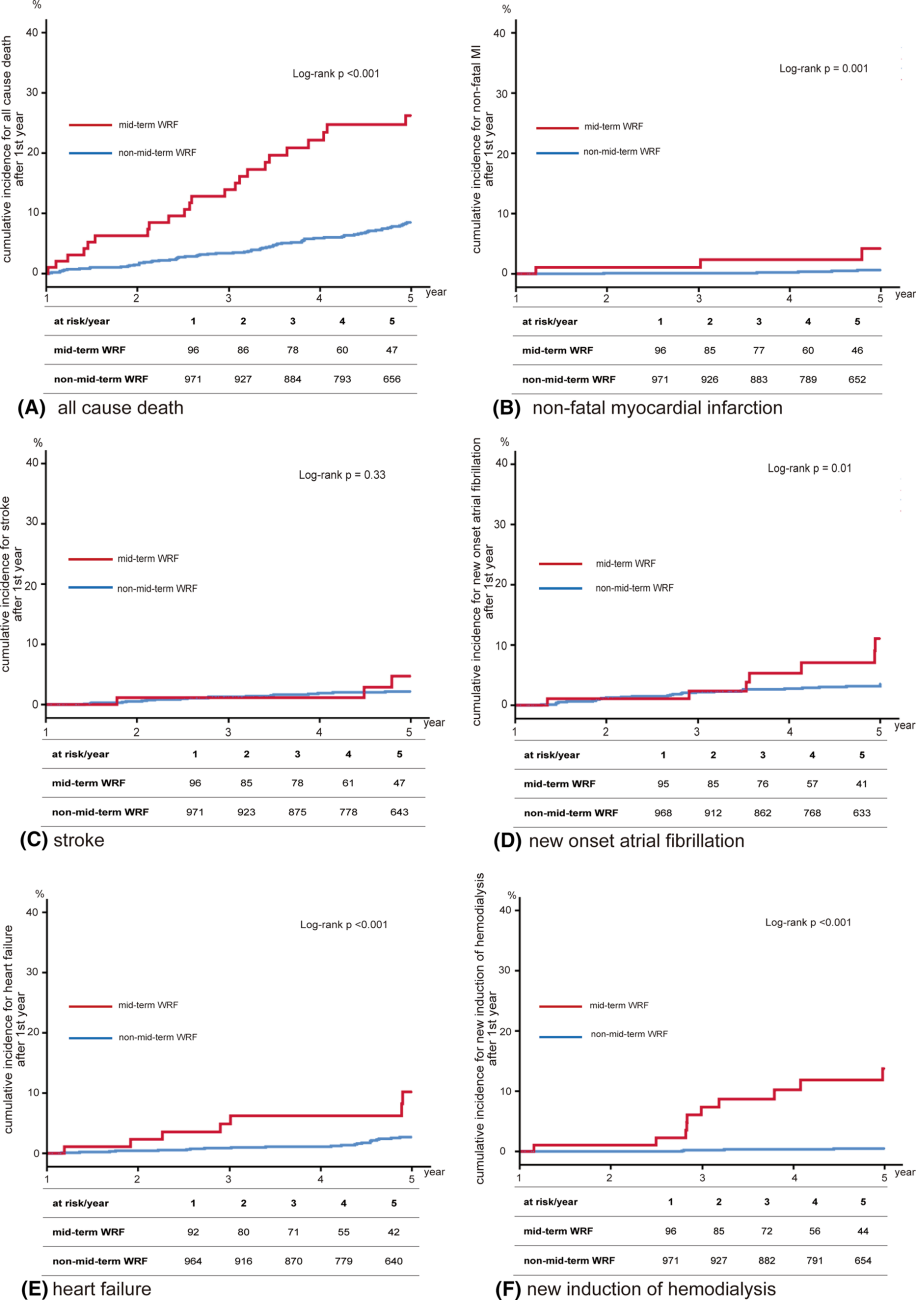
Cumulative incidence for any outcome according to WRF. **a** Death; **b** non-fatal myocardial infarction; **c** stroke; **d** new onset of atrial fibrillation; **e** heart failure; **f** new induction of hemodialysis. The incidences of death, non-fatal myocardial infarction, new-onset atrial fibrillation, future heart failure, and new induction of hemodialysis were significantly higher in WRF patients compared to non-WRF patients. *WRF* worsening renal failure, *MI* myocardial infarction

The incidences of MACE, death, and non-fatal MI were significantly higher in mid-term WRF patients compared to non-mid-term WRF patients in the first year post-PCI (26.7% vs. 9.7%, *p* < 0.001; 23.7% vs. 7.7%, *p* < 0.001; and 3.1% vs. 0.5%, *p* = 0.001, respectively). The incidence of stroke, however, was not significantly different (3.1% vs. 2.0%, *p* = 0.33). Furthermore, the incidence of new-onset AF, future heart failure, and new induction of hemodialysis were remarkably higher in mid-term WRF patients than in non-mid-term WRF patients (7.3% vs. 3.2%, *p* = 0.01; 7.5% vs. 2.3%, *p* < 0.001; 10.3% vs. 0.4%, *p* < 0.001, respectively) (Figs. 2, 3).

**Fig. 4:**
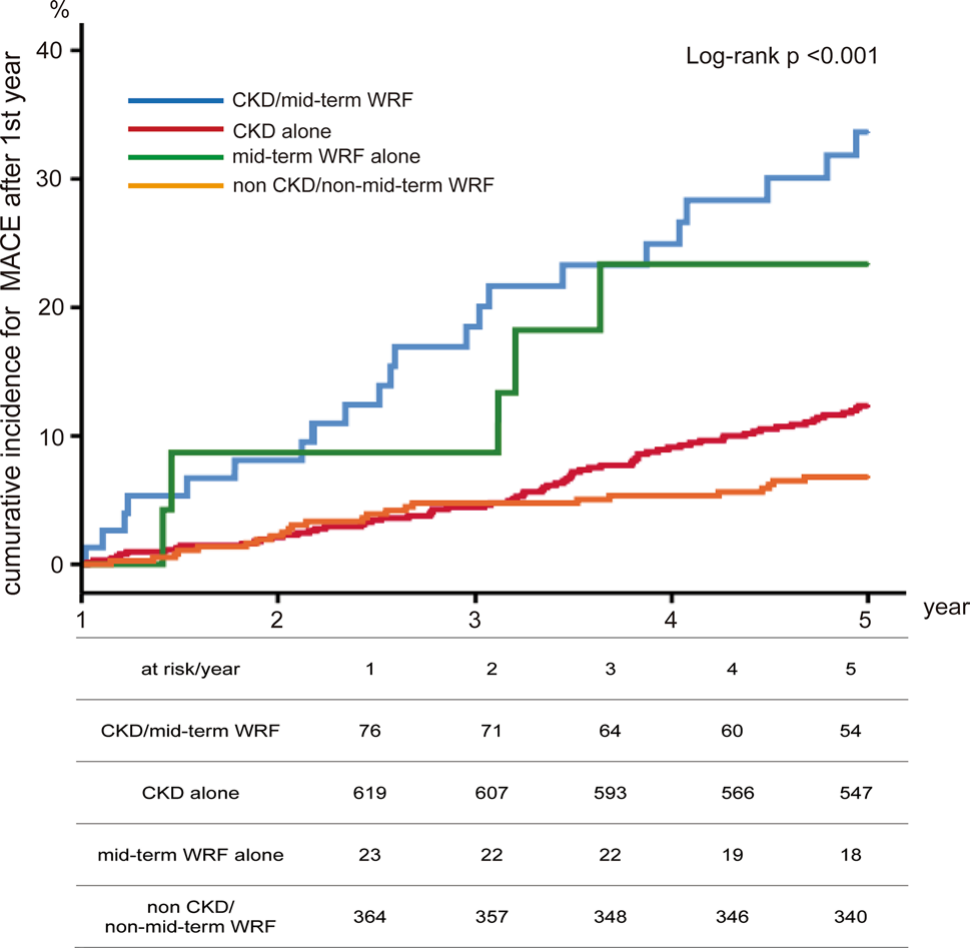
Cumulative incidence for MACE rate according to mid-term WRF and CKD. The incidence of MACE was significantly greater in CKD with mid-term WRF group compared to patients with only CKD, only mid-term WRF, and patients with neither CKD nor mid-term WRF. *MACE* major adverse cardiac event, *WRF* worsening renal function, *CKD* chronic kidney disease

**Fig. 5:**
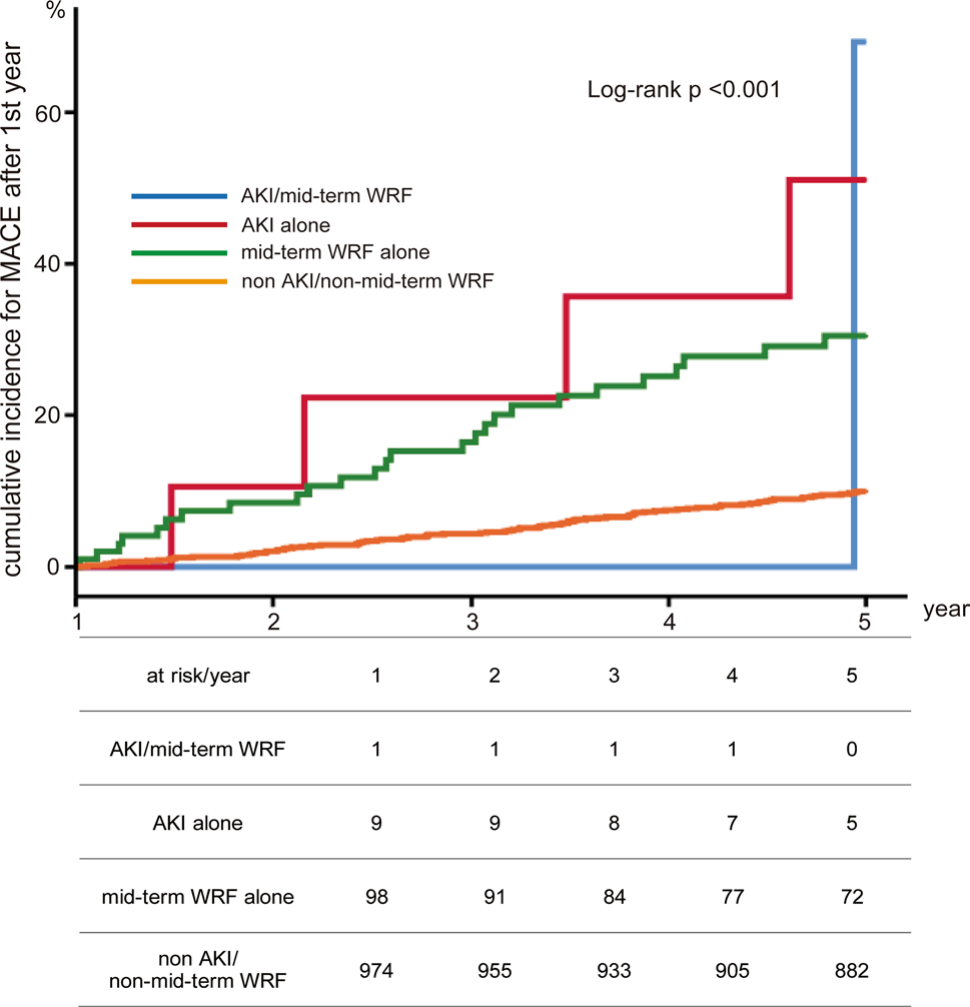
Cumulative incidence for MACE rate according to mid-term WRF and AKI. The incidences of MACE in patients AKI with mid-term WRF was significantly higher compared to patients with only AKI, mid-term WRF only, patients with neither AKI nor mid-term WRF group. *MACE* major adverse cardiac event, *WRF* worsening renal function, *AKI* acute kidney injury

When we evaluated CKD with mid-term WRF (*n* = 77), the incidence of MACE was significantly greater compared to patients with only CKD (*n* = 620), only mid-term WRF (*n* = 24), and patients with neither CKD nor mid-term WRF (*n* = 365) (28.6% vs. 11.6% vs. 20.8% vs. 6.6%, *p* < 0.001) (Fig. 4). Similarly, when we evaluated AKI with mid-term WRF (*n* = 2), the incidence of MACE was significantly higher compared to patients with only AKI (*n* = 10), mid-term WRF only (*n* = 99), patients with neither AKI nor mid-term WRF group (*n* = 975) (50.0% vs. 40.0% vs. 26.3% vs. 9.4%, *p* < 0.001) (Fig. 5). Multivariate Cox regression analysis revealed mid-term WRF as a strong predictor of MACE (hazard ratio: 2.50, 95% confidence interval 1.57–3.98, *p* < 0.001) (Table 2).

**Table 2:** Cox regression analysis

	Univariate Cox			Multivariate Cox		
	Hazard ratio	95% CI	*p*	Hazard ratio	95% CI	*p*
Age	1.07	1.05–1.09	< 0.001	1.07	1.04–1.09	< 0.001
Males	1.35	0.81–2.16	0.21	2.01	1.21–3.35	0.007
Body mass index	0.93	0.88–0.98	< 0.007	0.97	0.91–1.03	0.29
WRF	3.08	2.01–4.73	< 0.001	2.50	1.57–3.98	< 0.001
Chronic kidney disease	1.88	1.22–2.80	0.002	1.19	0.77–1.83	0.45
Congestive heart failure	1.79	1.15–2.78	0.009	1.07	0.66–1.73	0.79
Cerebral infarction	2.81	1.78–4.42	< 0.001	2.11	1.30–3.41	0.002
AF	1.98	1.26–3.11	0.003	1.26	0.77–2.06	0.37
Diabetes mellitus	1.67	1.17–2.38	0.005	1.78	1.23–2.57	0.002
Dyslipidemia	0.70	0.49–1.00	0.05	0.82	0.56–1.19	0.30
Hypertension	1.50	0.96–2.35	0.07			
Peripheral artery disease	1.64	0.99–2.70	0.05			
Chronic obstructive pulmonary disease	2.31	1.12–4.56	0.02			
Carotid artery stenosis	0.58	0.16–1.83	0.36			
Prior coronary artery bypass graft	1.69	0.99–2.90	0.06			
Old myocardial infarction	1.50	1.03–2.17	0.03			
Multi-vessel coronary disease	1.29	0.90–1.84	0.16			
ST-elevation MI	0.99	0.66–1.48	0.94			
Non-ST-elevation MI	0.77	0.28–2.07	0.60			
Unstable angina pectoris	1.10	0.63–1.91	0.75			
Hemoglobin	0.99	0.95–1.04	0.76			
Blood urea nitrogen	1.05	1.03–1.07	< 0.001			
Creatinine	1.35	1.14–1.60	< 0.001			
eGFR	0.98	0.97–0.99	< 0.001			
Left ventricle dysfunction	2.20	1.35–3.59	0.002			
Total contrast volume	1.00	0.99–1.00	0.07			

*MI* myocardial infarction, *CABG* coronary artery bypass graft, *AF* atrial fibrillation, *eGFR* estimated glomerular filtration rate

## Discussion

To summarize our study, (1) the incidence of mid-term WRF after PCI is 9.3%, (2) the incidence of MACE, death, MI and future heart failure were significantly higher in the mid-term WRF group, (3) the incidence of new-onset AF was remarkably higher in mid-term WRF, (4) hemodialysis use was significantly greater in mid-term WRF group, and (5) mid-term WRF adversely affects MACE, CKD and AKI.

In previous studies, the incidence of WRF during hospital stay was 6.3–19.8% after PCI [2–6], however, WRF in the acute phase was defined differently by each study. For example, some author defined WRF as a 25% reduction in eGFR compared to admission baseline, while others defined WRF as an increase in serum creatinine levels ≥ 0.3 mg/dL. WRF during hospital stay increased the risk of death, heart failure, cardiac shock, and stroke [3]. Goldberg et al. found that WRF is a powerful and independent predictor of 1-year mortality in addition to hospital death [5]. Further study showed that 4-year mortality was significantly higher in patients who developed WRF [4]. In our study, we defined mid-term WRF as an increase in creatinine ≥ 0.3 mg/dL and the 1-year time point after PCI, and found the incidence of WRF to be similar to previous studies.

In mid-term WRF patients, the incidence of MACE, death and MI were significantly higher. Latchamsetty et al. [6] assessed whether in-hospital WRF, either transient or sustained, is an independent risk factor for 6-month mortality in patients admitted with acute coronary syndrome. In their study, a return to baseline kidney function by the time of discharge did not protect the risk for mortality. As previous data showed, WRF is associated with worsening prognosis in patients with acute MI. However, attention should be paid to the evidence that WRF includes many risk factors for mortality: preexisting CKD, diabetes, left ventricle systolic function, peripheral artery disease, and so on. On the other hand, Goldberg et al. found a striking association between the occurrence of WRF during hospitalization and both in-hospital and 1-year mortality. In their study, patients with stable renal function showed a significant increase in mortality associated with reduced baseline renal function. More importantly, in patients developing WRF, the prognosis was equally dismal regardless of their baseline renal function [5]. From our study, continuous renal insufficiency after PCI also influenced prognosis, and is an independent predictor of MACE. Several studies have indicated that WRF is also related to longer hospital stays, higher rate of readmission, increased long-term mortality and activities of daily living decline [7–11]. Metra et al. reported that WRF is a frequent finding in patients hospitalized for acute heart failure and is associated with poor prognosis [12]. However, there is evidence to suggest that WRF does not adversely affect prognosis if it is a transient change. Aronson et al. have shown that transient WRF is frequent among patients with acute heart failure, but these patients appear to have a better outcome compared to persistent WRF patients [10]. In the study, persistent WRF predicted increased 6-month mortality. Taken in consideration with our findings, this evidence shows that clinicians need to be mindful that chronic deterioration of renal function affects not only for patients with acute heart failure, but also for patients after PCI. In addition, our study found that mid-term WRF affects long-term prognosis up to 5 years after PCI, whereas other studies had only conducted short-term follow-up.

There are no existing reports examining the relationship between WRF and AF. Basic experiments have reported that, in the heart failure model with frequent ventricular stimulation, myocardial fibrosis is more strongly induced in atrial muscle than in ventricular muscle, and tissue angiotensin II concentration is also higher in atrial muscle than in ventricular muscle [13]. As previous studies show, WRF can reflect venous congestion, which was largely demonstrated by an invasive study revealing a virtually linear relationship between creatinine and central venous pressure. Venous congestion and fluid overload due to WRF lead to increasing renal pressure, and activate the neurohormonal system (i.e., the renin–angiotensin–aldosterone system or arginine vasopressin system) [14, 15], and may be a trigger for AF development.

There are few reports about the relationship between WRF and hemodialysis or chronic renal function deterioration. In diabetes patients, the elderly, and patients with CKD, medial membrane calcinosis is common, and is associated with increased pulse wave velocity, elevated pulse pressure and systolic hypertension. As with many aspects of cardiovascular disease in CKD patients, there is a lack of knowledge regarding the vascular calcification process in early stages of CKD. However, there is increasing evidence that abnormal bone mineralization (occurring early in CKD), and vascular calcification are linked [16]. Mid-term WRF may be an early phase of chronic renal insufficiency, when atherosclerotic changes have already begun.

The association between cardiovascular disease and CKD were extensively explored [1, 16, 17], and other studies have documented renal insufficiency as an independent predictor of both short- and long-term cardiovascular morbidity and mortality [18]. Aoki et al. defined WRF as an increase in serum creatinine levels ≥ 0.3 mg/dL above admission baseline, and they reported that, among patients with eGFR ≥ 45 mL/min/1.73 m^2^, a significantly higher mortality rate was observed those who also had WRF [2]. Yagi et al. reported that CKD was associated with multivessel coronary artery disease, and the risk of cardiovascular event was threefold higher in the group with multivessel coronary artery disease and CKD [19]. In our study, we found 5-year prognosis after PCI was worse if the patients had CKD. Furthermore, the incidence of WRF worsened prognosis similarly to CKD, and when patients had both CKD and WRF, prognosis was significantly worse. Thus, kidney function deterioration influences prognosis, not only acutely but also in mid-term phase. Moreover, when we evaluated AKI and mid-term WRF, the incidence of MACE was significantly higher in mid-term WRF alone and AKI alone than in patients without either. Previous studies reported that AKI also increase mortality and worsens renal function [20], which is supported by our findings.

Our study exhibits potential limitations. First, there is no consensus for the definition of WRF, so we defined mid-term WRF based on existing studies. WRF was originally used when discussing heart failure but has recently become a term with broader application. Furthermore, in this study, we only used serum creatinine and not eGFR to determine if a patient had WRF. Previous studies have defined chronic kidney function impairment using eGFR, so evaluation of eGFR may have been pertinent. In our study, some people have used creatinine data as an indicator of renal function, while others have used eGFR, and more large number of patients had a data of creatinine. If we use eGFR as an indicator of kidney function, the target patient group is different, and different result may be obtained.

Second, we did not have serum creatinine data for 418 patients (27.2%) at 1 year after PCI. Of these, about 60% (253 patients) also showed signs of CKD. Further follow-up of these CKD patients may have led to other significant findings. It was also a limitation that we did not have proteinuria data. Compared with negative proteinuria, trace urine protein on dipstick tests was significantly associated with an increased risk of all-cause mortality and cardiovascular mortality at eGFR levels of 90–104 mL/min/1.73 m^2^, with similar associations for other eGFR levels [21]. Furthermore, the risk of end-stage kidney disease increased progressively with the levels of proteinuria on dipstick tests: the adjusted hazard ratio was 2.5 times in ± or 1 + group, 38 in 2 + group compare with non-proteinuria (in the patients whose eGFR 60–74 mL/min/1.73 m^2^) [22]. The effect of proteinuria on the prognosis of mid-term WRF requires additional study.

Third, the relationship between heart failure and renal dysfunction remains to be addressed. This study evaluated the long-term effects of mid-term WRF on cardiovascular events, heart failure, new induction of hemodialysis, and other factors. However, it is known that CKD is a strong predictor of advanced chronic heart failure [14], the risk of cardiovascular events increases progressively with CKD [1], and WRF may be associated with the worsened outcomes of cardiovascular death or heart failure hospitalization in cases of heart failure with or without preserved ejection fraction [7]. Although we adjusted the impact of mid-term WRF and heart failure using multivariate Cox regression, other factors may have influenced our results.

In conclusion, mid-term WRF after PCI adversely affected MACE, and future hospital admission due to heart failure and new-onset AF. Furthermore, we showed that WRF worsens not only the short-term prognosis, but long-term prognosis as well. It is also important to note that, patients whose kidney functions were already affected by AKI or CKD had worsened prognoses when combined with WRF. Thus, it is necessary for clinicians to take measures to prevent the impairment of renal function after PCI.

## Abbreviations

AF: Atrial fibrillation
AKI: Acute kidney injury
CABG: Coronary artery bypass graft
CKD: Chronic kidney disease
eGFR: Estimated glomerular filtration rate
MACE: Major adverse cardiac event
MI: Myocardial infarction
PCI: Percutaneous coronary intervention
WRF: Worsening renal function
